# Notes and News

**Published:** 1897-04-17

**Authors:** 


### NOTES AND NEWS.

(Collected and Communicated.)

The Duke of Northumberland has given £100 to the proposed Commemoration Convalescent Home for Women in Surrey, and Mr. Peckover, Lord Lieutenant of Cambridgeshire, has contributed another £1,000 towards the new operating theatre at Addenbrooke's Hospital, Cambridge.

The subject of the Queen's Commemoration has somewhat upset the equilibrium of Glasgow. It had been proposed to enlarge the Royal Glasgow Infirmary as an act of commemoration, and the scheme apparently met with general approval. But differences of opinion have arisen as to whether the extension demands a new and larger site. Dr. MacGregor Robertson is of opinion that it does, whilst others contend that the free air space provided by the outlook over the Necropolis minimises the objection to the limited area of the site, and to the proximity to this burying ground. Whilst this vexed question is under discussion energetic persons with other interests in view are pushing different proposals for the commemoration.

The Bishop of Chichester recently performed the opening ceremony of a new convalescent home at Rustington, Littlehampton The home has been built and endowed through the munificence of Mr. Harben, of Horsham, at an outlay of £70,000. Patients recommended by the North-West Convalescent Home Fund at Hampstead will have the pre-emption. The arrangement of the home is unique, being designed chiefly on the separate room principle. There are seventeen rooms with one bed, ten rooms with two beds, and two rooms with four beds. The brick and wood work utilised are also out of the common. The home is in the Georgian style of architecture. It stands back 250 ft. from the esplanade, and the west side of the building has an octagonal bay carried up two stories, and facing south is a sheltered terrace. A smoking-room, quiet room, reading-room and library, and invalids' day-room are provided. The whole building is lighted with electric light. The baths are provided with salt and fresh water. The administration block is separate from the patients' block, communicating with it by corridors.

A full account of Dr. Koch's latest tuberculosis antitoxin, which appears in the *Deutsche Medicinsche Wochenschrift*, is attracting much attention.

Madame Charcot has presented the pension of 2,000 francs awarded to her by the State to the Faculty of Medicine of Paris, to be applied to the assistance of widows and orphans of medical men connected with it.

The committee of the London Hospital has received an anonymous gift of £1,000 for the endowment of a bed in the hospital. The Skinners' Company has sent to St. George's Hospital a donation of 20 guineas.

The Japanese war-ship, "Fuji," in the Tilbury Docks, is to be thrown open to the public on Easter Monday, when one shilling will be charged to each visitor, for the benefit of the Seamen's Hospital.

The regulations of the Board of Health in New South Wales are so strict in regard to supervision of dairies that a number of dairymen are protesting, whilst others have given up the trade altogether. It is a matter of congratulation that the suppliers of dairy produce for exportation are willingly complying with the regulations.

Mr. Thomas Hall, of 2, Raymond Buildings, Gray's Inn, has bequeathed £5,000 in equal shares to the following institutions: The City of London Truss Society, the Governesses' Institution, the Royal National Lifeboat Institution, the Royal Society for the Prevention of Cruelty to Animals, the Royal Society for the Protection of Women and Children, the Royal Free Hospital, the Hospital for Sick Children, Great Ormond Street, the Central London Ophthalmic Hospital, the Cancer Hospital, and the Royal Sea Bathing Infirmary.

We regret to learn that for some time past the Regius Professor of Medicine at Oxford, Sir Henry Acland, has been very much out of health. He is now staying at Malvern, where, we trust, he will soon regain his strength. Sir Henry Acland's absence from Oxford is a matter of much regret. His exceptional social and intellectual qualities render his house a centre for pleasant intercourse not easily equalled, and where the youngest student to the most learned of his colleagues turn for the wise council and ready sympathy they never fail to receive at its owner's hands.

The London Medical Mission continues to do good. As is pointed out in the annual report, no mission workers can better ascertain the real needs of the poor than the doctors and nurses who are continually in their homes, and who see things as they are; and as it is the experience of all who work amongst the poor that the most needy are the last to make their wants known, the charitable are greatly indebted to such agencies as the London Medical Mission, which devotes itself to securing a true knowledge of the circumstances of the poor. The mission needs support for its many branches of useful work. It has a general fund, and special funds to maintain an invalid kitchen, a convalescent home, and a holiday home, to all or any of which contributions can usefully be sent.

